# The role of thoracic consolidative radiotherapy in the setting of immunotherapy in extensive stage small cell lung cancer

**DOI:** 10.1177/17588359231192399

**Published:** 2023-08-24

**Authors:** Saurav Verma, Sympascho Young, Alexander V. Louie, David Palma, Daniel Breadner

**Affiliations:** Division of Medical Oncology, Department of Oncology, Schulich School of Medicine & Dentistry, Western University, London, ON, Canada; London Regional Cancer Program at London Health Sciences Center, London, ON, Canada; Division of Radiation Oncology, Department of Oncology, Schulich School of Medicine & Dentistry, Western University, London, ON, Canada; London Regional Cancer Program at London Health Sciences Center, London, ON, Canada; Division of Radiation Oncology, Department of Oncology, Schulich School of Medicine & Dentistry, Western University, London, ON, Canada; Department of Radiation Oncology, University of Toronto, Toronto, Canada; Division of Radiation Oncology, Department of Oncology, Schulich School of Medicine & Dentistry, Western University, London, ON, Canada; London Regional Cancer Program at London Health Sciences Center, London, ON, Canada; Division of Medical Oncology, Department of Oncology, Schulich School of Medicine & Dentistry, Western University, A3-913 800 Commissioners Road East, London, ON N6A5W9, Canada; London Regional Cancer Program at London Health Sciences Center, London, ON, Canada

**Keywords:** extensive stage small cell lung cancer, immunotherapy, SCLC, thoracic radiotherapy, TRT

## Abstract

The improvement in treatment strategies and outcomes in small cell lung cancer (SCLC) has lagged behind other cancers. The addition of immune checkpoint inhibitors (ICIs), durvalumab and atezolizumab, to the platinum-based chemotherapy in frontline setting has improved the survival in extensive stage SCLC, (ES-SCLC), albeit modestly, and is now the new standard of care. Prior to advent of immunotherapy into the therapeutic armamentarium in ES-SCLC, consolidative thoracic radiotherapy (TRT) was associated with improved thoracic control and survival outcomes. In the era of ICIs, the role of TRT is not well defined, chiefly because TRT was not incorporated in any immunotherapy trials, secondly due to concerns regarding the increased risks of pneumonitis, and finally uncertain magnitude of benefit with this combined approach. In principle, radiation can increase in the immunogenicity of tumor and hence the activity of immune checkpoint blockade, thereby increasing efficacy both locally and distantly. Such an approach has been promising in non-small cell lung cancer with ICIs improving outcomes after concurrent chemoradiation, but remains unanswered in ES-SCLC. It is, thus, possible that the modest improvement in survival by addition of ICIs to chemotherapy in ES-SCLC can be further improved by the incorporation of consolidative TRT in selected patients. Several early phase trials and retrospective studies have suggested that such an approach may be feasible and safe. Prospective trials are ongoing to answer whether adding radiation therapy to chemoimmunotherapy will improve outcomes in ES-SCLC.

## Introduction

The last decade has seen a few improvements in the outcomes for patients with extensive stage small cell lung cancer (ES-SCLC) with the use of consolidative thoracic radiotherapy (TRT), prophylactic cranial irradiation, and immunotherapy. Prior to the evidence for incorporation of immune checkpoint inhibitors (ICIs), platinum-based doublet chemotherapy (CHT) was the standard of care for treating ES-SCLC.^1,2^ Consolidative thoracic radiotherapy entered the treatment paradigm with two phase III trials showing improvement in thoracic control and survival outcomes in a sub-group of patients with ES-SCLC who had a good response at the metastatic sites.^3,4^ The chemoimmunotherapy trials did not permit consolidative TRT in the experimental or control arms. The evidence is lacking for its use in combination with immunotherapy although some early phase trials have established safety. There is a lack of literature about the role of TRT in the era of upfront chemoimmunotherapy. Progress has been made in stage III non-SCLC with immunotherapy improving outcomes after concurrent chemoradiation. Similar approaches of combining consolidative TRT and immunotherapy are promising and being tested in SCLC in prospective randomized trials. In this review we discuss ICI’s and TRT in ES-SCLC, the rationale and current literature on their combination, and the prospects of these modalities in combination.

## Thoracic radiotherapy in ES-SCLC

Although ES-SCLC is highly sensitive to chemotherapy, 80–90% of patients have residual intrathoracic disease. While systemic progression is the main pattern of progression, a vast majority develops intrathoracic relapse or progression within a year of diagnosis, making intrathoracic tumor control a major difficulty.^3^ Uncontrolled thoracic disease progression can cause significant morbidity and complications such as superior vena cava (SVC) obstruction, lung collapse, post-obstructive pneumonia, and respiratory distress. This provided rationale that by improving thoracic local control in patients with ES-SCLC, consolidative TRT may also lead to decreased morbidity and improved survival.

A literature review in the 1980s, of non-randomized and randomized trials, first showed that TRT decreases the rates of thoracic failure, though it did not improve response rates or survival.^5^ Several other retrospective analyses also suggested that TRT in combination with CHT was associated with an improvement in long-term survival (Table 1).

**Table 1.** Studies for thoracic radiotherapy (TRT) before the advent of immunotherapy in treatment paradigm.

Study	Phase	Intervention	Median OS	Median PFS	Local progression	Toxicity
Jeremic *et al*.^4^ *N* = 210	III	Arm A – TRT (concurrent) +chemotherapy Arm B – chemotherapy alone	17 *versus* 11 months 2-year OS, 38% *versus* 28% 5-year OS, 9.1% *versus* 3.7%	5-year LRFS, 20% and 8.1%	Median LRFS, 30 *versus* 22 months	Acute high grade toxicity higher in Arm B
Slotman *et al*.^3^ *N* = 498	III	TRT (sequential) after chemotherapy response *versus* chemotherapy alone	8 *versus* 8 months 1-year OS, 33% *versus* 28% 2-year OS, 13% *versus* 3%	4 *versus* 3 months 6-month PFS 24% *versus* 7%	Isolated thoracic progression, 19.8% *versus* 46%	G 3–5 AEs – 10.5% *versus* 7.3%
Gore *et al*.^6^ *N* = 97	II	PCI + cRT to thorax plus extracranial metastases *versus* PCI alone	13.8 *versus* 15.8 months 1-year OS, 50.8% *versus* 60.1%	4.9 *versus* 2.9 months	Locoregional disease at first failure, 62.5% *versus* 25.8%	G 3–5 AEs – 36.4% *versus* 23.8%
Yee *et al.*^7^ *N* = 32	II	TRT (sequential)	8.3 months	4.2 months	–	G 2 esophagitis – 56.3%
Zhu *et al*.^8^ *N* = 119	Retrospective	TRT (sequential) + chemotherapy *versus* chemotherapy alone	17 *versus* 9.3 months 2-year OS, 35% *versus* 17% 5-year OS, 7.1% *versus* 5.1%	10 *versus* 6.2 months 2-year PFS, 12.6% *versus* 7.2% 5-year PFS, 6.3% *versus* 5.4%	Local relapse, 35% *versus* 52.1%	G 2-3 pneumonitis 6.7% with TRT G 2-3 esophagitis 21.6% with TRT
Li-Ming *et al.*^9^ *N* = 306	Retrospective	TRT (sequential) + chemotherapy *versus* chemotherapy alone	2-year OS, 21.4% *versus* 10.3%	2-year PFS 7.7% *versus* 4.6%	Local relapse, 10.2% *versus* 17% Local control at 2 years 34.5% *versus* 6.3%	G 2–3 pneumonitis and grade 2 esophagitis higher with TRT
Giuliani *et al*.^10^ *N* = 19	Retrospective	TRT (sequential)	14 months 1-year and 2-year OS, 58% and 14%	9 months 1-year PFS, 26%	Locoregional failure at 1 year, 26%	No clinical pneumonitis
Tian *et al.*^11^ *N* = 14,367	Retrospective analysis of national database	TRT + chemotherapy *versus* chemotherapy alone	12.1 *versus* 8.2 months 1-year OS, 50.5% *versus* 28.5% 5-year OS, 7.6% *versus* 2.0%	–	–	–

AEs, adverse effects; CI, confidence interval; cRT, consolidative radiotherapy; G, grade; HR, hazard ratio; LRFS, local recurrence free survival; OS, overall survival; PCI, prophylactic cranial irradiation; PFS, progression free survival; SBRT, stereotactic body radiotherapy; TRT, thoracic radiotherapy.

In the 1990s, role of radiation therapy (RT) in ES-SCLC was not well defined. Jeremic *et al.* conducted the first trial that showed survival benefit of consolidative RT. This was a randomized trial that evaluated TRT in subsets of patients with various degrees of response in thoracic and distant sites to find a subgroup most likely to benefit from TRT. Patients with ES-SCLC were treated with three cycles of CHT (cisplatinum/etoposide (PE)) and those with a complete response (CR) at both the local and distant (CR/CR) or a partial response (PR) at the local site and CR at the distant sites (PR/CR) received thoracic accelerated hyperfractionated RT (54 Gy in 36 fractions BID) with PE followed by two–four cycles of PE. All patients also received prophylactic cranial irradiation (PCI). Patients with CR at distant sites and at least a PR in the thorax had significantly better 5-year overall survival (OS) when they received consolidative TRT compared to those that received chemotherapy alone (9.1% *versus* 3.7%; *p* = 0.041).^4^ Notably, about 90% of all patients in this study had low metastatic burden with 1–2 metastases. It can be commented that this study showed survival benefit of aggressive (high total tumor dose of 54 Gy in 36 fractions, 1.5 Gy BID) consolidative/concurrent TRT in a selected patient population with good performance status, excellent response after chemotherapy (with CR at distant sites) and low metastatic burden.

In 2015, results from Chest Radiotherapy Extensive-Stage Trial (CREST) phase III randomized trial demonstrated a 10% 2-year improvement in OS for patients who responded to chemotherapy with subsequent TRT. In this trial, patients with any response after chemotherapy were randomly assigned to receive either TRT (30 Gy in 10 fractions) or no TRT. All patients also received PCI. The primary end point, OS at 1 year, was not significantly different between the two groups (33% TRT *versus* 28% chemotherapy alone; *p* = 0.066). However, 2-year OS was 13% (95% confidence interval, 9–19) *versus* 3% (95% CI, 2–8; *p* = 0.004) and 6-month progression-free survival (PFS) was 24% (95% CI, 19–30) *versus* 7% (95% CI, 4–11; *p* = 0.001), showing significant benefit with TRT. TRT was not associated with any severe toxic effects. On subgroup analysis, it was found that patients with residual intrathoracic disease after chemotherapy had significant improvements in OS, PFS, and reduced risk of intrathoracic progression if they undergo consolidative TRT. Patients with complete resolution of intrathoracic disease did not appear to have the same benefits, though the trial was not powered to study this smaller subgroup (only 11% of patients).^12,13^ Secondary analysis also found that patients with two or fewer metastases had better outcomes.^14^ This trial established that in patients with ES SCLC who respond to CHT, TRT should be considered in addition to PCI.^3^ Notably, in CREST, consolidative TRT was not concurrent and less intensive (hypofractionated, 30 Gy in 10 fractions) compared to the study by Jeraemic *et al.*

A systematic review with meta-analysis of the above two trials (Jeremic *et al.* and CREST) showed that TRT was associated with improved OS [Hazard ratio (HR), 0.81; 95% CI, 0.69–0.96; *p* = 0.014] and PFS (HR, 0.74; 95% CI, 0.64–0.87, *p* < 0.001). The grade 3 or higher esophageal toxicity was 6.6% in the TRT arm and 0% without TRT (*p* < 0.001).^15^ Another systemic review reported similar conclusions.^16^

The NRG Oncology RTOG 0937 trial, a phase II randomized study, explored a different but related question. RTOG 0937 assessed whether PCI plus consolidative RT (radiation therapy to the locoregional thoracic disease and 1–4 active sites of metastases after CHT) would improve OS compared with PCI alone for patients with ES-SCLC, in a select population of patients with 1–4 oligometastases. This study used a dose/fractionation scheme of 45 Gy in 15 fractions to the thorax and sites of metastasis, though lower doses of 30–40 Gy was allowed if there was difficulty meeting dose constraints. While consolidative radiation initially delayed progression (with a 3-month rate of progression of 14.5% *versus* 53.3% in the PCI alone arm), the study ultimately failed to meet its primary endpoint of 1-year OS and was closed early due to futility.^6^

## Immunotherapy in ES-SCLC

Combining immunotherapy with CHT upfront is now the standard of care in ES-SCLC. Two phase III randomized control trials, IMpower 133 and CASPIAN, have demonstrated the benefit of adding atezolizumab and durvalumab [programed death-ligand 1 (PD-L1) inhibitors] respectively to chemotherapy, followed by ICI maintenance, in the first line setting.^1,2^ However, this benefit is modest with approximately 2 months median OS advantage (Table 2).

**Table 2.** Randomized clinical trials in first line setting for immunotherapy in ES-SCLC.

Trial	Phase	Intervention	Primary endpoint	Results
IMpower133^2^	III	Carboplatin + etoposide + atezolizumab or carboplatin + etoposide + placebo	PFS and OS	median OS − 12.3 and 10.3 months (HR, 0.76; 95% CI, 0.60–0.95; *p* = 0.0154) median PFS − 5.2 *versus* 4.3 months (HR, 0.77; 95% CI, 0.62–0.96; *p* = 0.02)
CASPIAN^1^	III	Arm A – Cisplatin/carboplatin + etoposide (EP) +durvalumab; Arm B – EP + tremelimumab + durvalumab; Arm C – EP	OS – Arm A vs arm C OS – Arm B vs arm C	median OS (Arm A *versus* arm C)− 12.9 versus 10.5 months (HR, 0.71; 95% CI, 0.60–0.86; *p* = 0.0003]
KEYNOTE 604^17^	III	Cisplatin/carboplatin + etoposide (EP) +pembrolizumab or EP + placebo	OS and PFS	median OS – 10.8 *versus* 9.7 months (HR, 0.76; 95% CI, 0.63–0.93) median PFS – 4.8 *versus* 4.3 months (HR, 0.70; 95% CI, 0.57–0.85)
ECOG-ACRIN EA5161^18^	II	Cisplatin/carboplatin + etoposide (EP) +nivolumab or EP	PFS	median PFS − 5.5 *versus* 4.6 months (HR, 0.65; 95% CI, 0.46–0.91; *p* = 0.012)
Reck *et al.*^19^	II	Arm A – Carboplatin/paclitaxel (PT) with phased ipilimumab Arm B – PT with concurrent ipilimumab Arm C – PT with placebo	immune-related (ir) PFS	Median irPFS (Arm A *versus* C) − 6.4 and 5.3 months (HR, 0.64; *p* = 0.03] No PFS or OS benefit
Reck *et al.*^20^	III	Cisplatin/carboplatin + etoposide (EP) +ipilimumab or EP with placebo	OS	median OS − 11.0 vs 10.9 months (HR, 0.94; 95% CI, 0.81–1.09; *p* = 0.3775)
CheckMate 451^21^	III	Nivolumab plus ipilimumab maintenance or placebo	OS	median OS − 9.2 *versus* 9.6 months (HR, 0.92; 95% CI, 0.75–1.12; *p* = 0.37)

CI, confidence interval; HR, hazard ratio; OS, overall survival; PFS, progression free survival.

In IMpower 133, patients were randomized to either atezolizumab plus CHT or placebo plus CHT, and then either maintenance atezolizumab or placebo. In an updated analysis, the median follow-up was 22.9 months; the median OS was 12.3 and 10.3 months with atezolizumab plus CHT and placebo plus CHT, respectively (HR, 0.76; 95% CI, 0.60–0.95; *p* = 0.0154). The benefit was irrespective of PD-L1 immunohistochemistry or blood-based tumor mutational burden (bTMB) status.^2,22^

In CASPIAN, there was a 1:1:1 randomization to durvalumab plus CHT, durvalumab plus tremelimumab plus CHT, or CHT alone. The primary endpoints were OS for durvalumab plus CHT *versus* CHT and for durvalumab plus tremelimumab plus CHT *versus* CHT. After a median follow-up of 39.4 months, durvalumab plus CHT showed an improved median OS *versus* CHT alone, 12.9 *versus* 10.5 months respectively, with a HR 0.71 (95% CI, 0.60–0.86; *p* = 0.0003). The 3-year OS rate was 17.6% *versus* 5.8%.^1,23^

KEYNOTE-604, a randomized placebo-controlled phase III trial analyzed pembrolizumab *versus* placebo in combination with CHT, followed by pembrolizumab *versus* placebo maintenance in patients with treatment-naïve ES-SCLC. Though the primary endpoint of PFS was met, median PFS of 4.5 *versus* 4.3 months in favor of pembrolizumab (HR 0.75, 95% CI 0.65–0.91; *p* = 0.0023), the co-primary endpoint of OS was not met despite a numerically improved median OS of 10.8 *versus* 9.7 months (HR 0.8, 95% CI 0.64–0.98; *p* = 0.0164). The 12-month PFS was 13.6% with pembrolizumab plus CHT and 3.1% with placebo plus CHT.^24^

A meta-analysis concluded that ICIs plus chemotherapy was associated with significantly improved PFS and OS. Both PD-1 inhibitors and PD-L1 inhibitors (HR, 0.73; 95% CI, 0.63–0.85) plus chemotherapy led to improvement in PFS and OS with no further increase in toxicity; however, CTLA-4 inhibitors did not improve survival and were associated with more toxicity. Patients with baseline brain metastases did not benefit with ICIs. The pooled overall response rates (ORR) were comparable between ICIs plus chemotherapy and chemotherapy alone.^25^

CheckMate 451 evaluated maintenance therapy with nivolumab plus ipilimumab for patients with ES-SCLC who did not progress on first-line chemotherapy. Although no new safety signals were seen, the primary endpoint of OS was not met.^21^

## Combining TRT and immunotherapy

After first line chemoimmunotherapy, approximately 70–75% of ES-SCLC patients have residual disease while around 75% of patients will have disease progression despite an initial response.^23^ The initial trials with TRT in the era of chemotherapy were an effort to improve intrathoracic disease control to improve patient survival outcomes. The IMpower 133 and CASPIAN trials did not include TRT. Despite both consolidative TRT and immunotherapy improving outcomes, the combination of the two has not been evaluated extensively (Table 3). The key concerns remain, firstly the safety of combining radiation therapy with immunotherapy in ES-SCLC; and secondly, specific questions pertaining to which patient population may benefit, optimal dose, timing, sites (local *versus* oligometastatic) and dose/fractionation schemes.

**Table 3.** Studies for thoracic radiotherapy (TRT) in the era of immunotherapy in ES-SCLC.

Study	Phase	Intervention	Median OS	Median PFS	Toxicity
Welsh *et al.*^26^ N-38	I	TRT + concurrent pembrolizumab	8.4 months	6.1 months	G4-5 AE − 0 G3 AEs − 6%
Perez *et al.*^27^	I/II	Chemotherapy followed by cTRT followed by ipilimumab + nivolumab for an year	11.7 months	4.5 months	⩾1 G3-4 irAEs – 52% G3 pulmonary irAEs − 19%
Pakkala *et al.*^28^	II	Arm A - durvalumab/ tremelimumab Arm B - durvalumab/tremelimumab + SBRT	2.8 *versus* 5.7 months	2.1 *versus* 3.3 months	The most common AEs were G1 in two arms.
Galuba *et al.*^29^	Retrospective	Chemoimmunotherapy with/without RT	–	–	No new safety signals.
Diamond *et al.* ^30^ N-20	Retrospective	CHT + immunotherapy + cTRT	16 months	6.7 months	G2 esophagitis − 5% No ⩾G2 pneumonitis, No ⩾G3-5 AEs
Gross *et al.*^31^ N-244	Retrospective	Chemoimmunotherapy with/without TRT	11 *versus* 9 months	–	–
Bruni *et al.*^32^ N-31	Retrospective	Chemoimmunotherapy with/without TRT	7.7 months	5.4 months	No difference in AEs between those who did or did not undergo TRT.

AEs, adverse effects; CI, confidence interval; cTRT, consolidative thoracic radiotherapy; G, grade; HR, hazard ratio; OS, overall survival; PFS, progression free survival; RT, radiotherapy; SBRT, stereotactic body radiotherapy; TRT, thoracic radiotherapy.

The rationale behind combining TRT and immunotherapy is the preclinical and clinical evidence showing synergy between them.^33^ The biological mechanism for the synergy of the combination of RT and ICIs is based on the immunomodulating effect of RT.^33,34^ Radiation can debulk tumor mass, lead to increased antigen presentation, promote T cell infiltration, and affect the immune milieu in tumor microenvironment. This can enhance response, at local and distant sites (abscopal effect), and lead to better outcomes.^35–37^ Although most data for abscopal effect comes from non-small-cell lung cancer (NSCLC), it has been observed in other cancers.^38^

Verma *et al.* analyzed the safety of combined immunotherapy and TRT (iRT) in patients from three phase I/II trials and found combined iRT to be safe in the short term (median follow-up was 6.9 months (range, 0.5–30.9 months).^39^ The safety of combining ICI and RT was tested in another phase I trial, which enrolled patients with ES-SCLC treated with induction CHT followed by concurrent pembrolizumab and TRT (45 Gy in 15 fractions, with simultaneous integrated boost to the gross tumor allowed to 52.5 Gy). The combination was safe with no grade 4–5 toxicities and only 6% of patients experiencing grade 3 events (median follow-up 7.3 months; range 1–13 months).^26^ The limitations of this study included short follow-up, patient heterogeneity (including NSCLC and SCLC, varying levels of baseline lung disease), treatment heterogeneity (varying fractionation schemes, prior thoracic RT, RT dose overlap, and immunotherapy agents (delivered sequentially and concurrently)). Due to heterogenous population, outcomes variables could not be interpreted. The ORR was 79%. There was no incidence of pneumonitis and 15% experienced grade 2 esophagitis.

In a retrospective analysis of patients with ES-SCLC (*N* = 20) treated with chemoimmunotherapy and consolidative TRT, the median OS was 16 months. The 6-month OS of 94.7% and 12-month OS of 77.5% was comparable to historical controls. Their toxicity included 0% grade 3 or more toxicity, 0% grade 2 pneumonitis, and 5% grade 2 esophagitis.^30^ In another small retrospective analysis by Galuba *et al.* the patients who received consolidative TRT showed no deterioration in pulmonary functions, and no severe toxicity was observed.^29^ Gross *et al.* examined the National Cancer Database (NCDB) in United States for patients treated with a combination of CHT and ICI at the time of diagnosis, and with and without TRT. A trend toward improved median OS from 9 to 11 months and the 2 year-OS was numerically higher at 18.1% with the addition of TRT vs. 12.0% with chemoimmunotherapy alone (*p* = 0.067).^31^

Perez *et al.* studied whether adding ipilimumab and nivolumab after TRT to residual primary and initially involved regional lymph nodes (30 Gy in 10 fractions) would improve outcomes for patients with ES-SCLC. In this phase I/II study, 21 patients were enrolled. The median PFS was 4.5 months (95% CI, 2.7−4.6%) and the median OS was 11.7 months (95% CI, 4.7–16.0%). Though PFS was not improved, OS was higher than historic controls. However, the toxicity was significant with 52% of patients having *a* ⩾ 1 possibly related grades 3–4 immune-related adverse events (irAEs); and grade 3 pulmonary and gastrointestinal irAEs were recorded in 19% and 24% of patients, respectively.^27^ This increased toxicity may largely be attributable to the immunotherapy component, as the toxicity profile was comparable to that of the ipilimumab/nivolumab arm following platinum-based chemotherapy in Checkmate 451.^37^

A phase II study evaluated durvalumab (D)/tremelimumab (T) *versus* immune-sensitizing stereotactic body radiation therapy (SBRT) to a selected tumor site (9 Gy in three fractions) followed by D/T until progression or a maximum of 12 months, in relapsed ES-SCLC. The co-primary endpoints of the study were ORR and PFS. The D/T combination with/without SBRT was safe; however, efficacy wasn’t improved. As a translational endpoint to study pharmacodynamic biomarkers for better selection of patients that could benefit from immunotherapy, the study evaluated lymphocyte subsets in paired peripheral blood samples and tumor infiltrating lymphocytes (TILs) on treatment biopsies (pre and post two cycles of chemoimmunotherapy). There was a significant increase in levels of activated circulating CD8+ *T* lymphocyte subset in peripheral blood and higher proportion of activated T cells in TILs from biopsy samples in responders.^28^

The current ASTRO guidelines recommend that for patients with ES-SCLC with a response to chemotherapy and immunotherapy and residual disease in the thorax, consolidative TRT (30 Gy in 10 fractions within 6–8 weeks) is conditionally recommended, and 45 Gy in 15 fractions can be considered for well selected fit patients.^40^ The strength of recommendation is conditional, and quality of evidence is based on expert opinion. National Comprehensive Cancer Network (NCCN) mentions that sequential TRT can be considered for selected patients, during or before maintenance immunotherapy, in the absence of clinical data.^41^ ESMO calls for shared decision making when using TRT after/during chemoimmunotherapy.^42^ The uncertainty underlying these recommendations likely reflects the fact that magnitude of benefit of TRT is unknown.

## Future

As SCLC enters the era of precision therapy, with the unraveling of genomic subtypes of SCLC, more will be known about which subgroups may benefit with various modalities.^43^ For example, Gay *et al.* found that SCLC variants with more inflamed signatures have a favorable response with the ICIs compared to dismal response in other less inflamed subtypes. It will be interesting to find whether addition of RT to the immunosuppressed variants can activate and modulate the immune profile, making them more responsive to ICIs. The optimal timing of ICIs and RT remains unanswered. The available literature suggests that newly recruited T cells can destroy tumor cells after being presented with tumor antigens, and as such either simultaneous or sequential administration of ICIs after RT may be a good strategy. Predictive biomarkers may also help in patient selection for benefit from immunotherapy and/or consolidative TRT.

Ultimately ongoing trials (Table 4) are needed to shed light on the feasibility and efficacy of RT with ICIs in ES-SCLC. Phase I RCTs, including PRIO and PICARES, are evaluating the safety of this approach.^44,45^ The Raptor trial (NRG-LU007) is a phase II/III trial evaluating consolidative RT in ES-SCLC with atezolizumab. It is currently accruing patients with a stable disease or a PR after initial systemic treatment. Patients will be randomized to receive either atezolizumab maintenance or consolidative RT to up to five sites (including thoracic and extra thoracic; liver, bone, CNS, etc.) followed by maintenance atezolizumab. The phase II portion will evaluate the PFS while phase III will evaluate OS.^46^

**Table 4.** Ongoing trials for thoracic radiotherapy (TRT) with immunotherapy in SCLC.

Study	Phase	Intervention	Primary endpoint
RAPTOR/NRG-LU007^46^ Active, recruiting	II/III	Consolidation radiation and atezolizumab vs atezolizumab alone	PFS OS
NCT02402920^47^ Active, not recruiting	I	cTRT with pembrolizumab	Safety
NCT04728230^44^ PRIO trial Active, recruiting	I	Chemoimmunotherapy, cTRT, olaparib, durvalumab	Safety and feasibility
TREASURE NCT04462276^48,49^	II	atezolizumab maintenance therapy with or without TRT	OS
PICARES NCT03971214^45^ Not recruiting	I	PD-1 inhibitors consolidation after standard first-line chemotherapy and radiotherapy	Safety, ORR

AEs, adverse effects; CI, confidence interval; cTRT, consolidative thoracic radiotherapy; G, grade; HR, hazard ratio; ORR, overall response rate; OS, overall survival; PFS, progression free survival; RT, radiotherapy; SBRT, stereotactic body radiotherapy; TRT, thoracic radiotherapy.

## Conclusion

Although TRT has been shown to improve outcomes in the pre-immunotherapy era, the applicability of that data in current practice is unknown. There are several early-phase studies in ES-SCLC suggesting that TRT is safe with chemoimmunotherapy. However, larger prospective trials with longer follow-ups are needed to answer the question definitively before this strategy can be recommended and routinely implemented. We need to find the appropriate population, dose, and timing of TRT to tip the scale and maximize benefit while minimizing toxicities. With evolving knowledge of genomic signatures embarking the era of precision therapy in SCLC, there is hope for further improving outcomes in ES-SCLC.

## Declarations

**Ethics approval and consent to participate:** Not applicable.

**Consent for publication:** Not applicable.

**Availability of data and materials:** Not applicable.
